# Factors associated with psychological distress among end-of-life care volunteers: a systematic review of quantitative and qualitative evidence

**DOI:** 10.1186/s12904-026-02201-1

**Published:** 2026-06-26

**Authors:** Lina Mebus, David J. Pedrosa, Anna J. Pedrosa

**Affiliations:** 1 Fraunhofer Institute for Applied Information Technology FIT, Sankt Augustin, Germany; 2 Medical Faculty, Department of Neurology, Marburg University, Marburg, Germany

**Keywords:** Palliative care, Hospice care, Volunteers, Psychological distress, Caregiver burden, Depression, Anxiety

## Abstract

**Background:**

Volunteers are integral to end-of-life care, providing emotional, spiritual, and practical support. However, they often face emotionally demanding situations with limited training and supervision compared to professionals. Given the limited and fragmented literature on psychological distress experienced by end-of-life volunteers, this systematic review aimed to synthesise existing quantitative and qualitative evidence to identify factors associated with psychological distress.

**Methods:**

We conducted a systematic literature review including qualitative and quantitative evidence. Five databases (MEDLINE, EMBASE, PsycINFO, Cochrane Database and Web of Science) were searched for original studies, complemented by citation and reference searches. Study quality was assessed using the Qualsyst tool. Quantitative findings were synthesised using an algorithm to evaluate evidence strength, and qualitative data were integrated through thematic meta-synthesis.

**Results:**

Twenty-six studies (20 quantitative and 6 qualitative studies) met inclusion criteria. Quantitative research examined 49 volunteer-related, 18 service-related, and one volunteer-patient-interaction-related factor associated with anxiety, death anxiety, depression, burnout, and/or perceived stress. Moderate-strength evidence indicated that death anxiety was negatively associated with better health and well-being but unrelated to age, volunteer experience, or training. Furthermore, depression was negatively associated with volunteer training. Qualitative evidence was scarce, but highlighted additional patient-, interaction-, and service-level mechanisms.

**Conclusion:**

This review identifies a small, methodologically diverse evidence base on factors associated with psychological distress in end-of-life care volunteers. Quantitative evidence suggests a potential protective association between training and depression, though substantial heterogeneity limits firm conclusions. Limited qualitative evidence revealed patient-, interaction- and service-level factors that are rarely captured quantitatively. Robust theory-guided longitudinal studies are needed to better understand distress and resilience in this under-researched group.

**Supplementary Information:**

The online version contains supplementary material available at 10.1186/s12904-026-02201-1.

## Introduction

As societies transform, so do the ways families care for their older and dying members. Increasingly, care of these particularly vulnerable individuals has become a shared responsibility within the wider community [1]. Volunteers have been central to this shift. They were key figures in the early palliative care movement [2] and remain vital contributors to end-of-life care services worldwide. Through emotional, spiritual, and practical support, volunteers complement the work of families and professionals alike and help ensure that care remains truly compassionate [3, 4].

Although the evidence base is limited, findings suggest that palliative care interventions that include volunteers may enhance family satisfaction and could be associated with prolonged patient survival [4]. In contrast, while volunteering is generally linked to better mental health outcomes for volunteers [5], little is known about the psychological challenges faced by volunteers in end-of-life care settings. In contrast, psychological distress among professional palliative care providers has been widely studied, with research showing that clinicians working in palliative care are at significant risk for burnout, compassion fatigue, and moral distress [6]. Furthermore, these professionals frequently experience emotional exhaustion, reduced empathy, and ethical tension due to prolonged exposure to suffering and organisational challenges [7].

End-of-life care volunteers receive less training than professionals [3, 8] but are routinely exposed to the same situations involving suffering, dying, and death. Although volunteering in this setting can be deeply meaningful and rewarding, it also entails relevant intrinsic and extrinsic challenges [9, 10]. While psychological burden has been examined as an important aspect of end-of-life care volunteers’ experiences in a systematic review and thematic synthesis [10], no comprehensive synthesis has yet specifically assessed factors that may influence psychological distress, drawing on both qualitative and quantitative evidence. However, understanding the factors associated with psychological distress in volunteers is crucial for sustaining their well-being and engagement. Insights in this area may also help identify volunteers at greater risk and inform the development of strategies for the prevention of psychological distress and its management.

This systematic mixed-methods review aims to close a research gap by identifying and synthesising factors associated with psychological distress in volunteers working in end-of-life care.

## Methods

### Design

This systematic review of quantitative and qualitative studies conducted in parallel syntheses is reported in accordance with the Preferred Reporting Items for Systematic Reviews and Meta-Analyses (PRISMA) guideline [11]. Reporting of the qualitative synthesis was additionally informed by the ENTREQ statement [12]. The review protocol was registered in PROSPERO in November 2024. The review question guiding this review was: ‘What factors are associated with psychological distress in end-of-life care volunteers?’ The review integrated quantitative and qualitative findings to provide a comprehensive overview of the factors contributing to psychological distress among end-of-life care volunteers.

### Eligibility criteria

We included studies that reported original empirical data on at least one factor associated with psychological distress in end-of-life care volunteers. Psychological distress is a multidimensional and variably conceptualised construct within the literature. The American Psychological Association defines psychological distress as “a set of painful mental and physical symptoms that are associated with normal fluctuations of mood in most people” [13]. In some individuals, psychological distress may also represent an early manifestation of clinically significant mental health conditions, including depressive and anxiety disorders, schizophrenia, somatic symptom disorders, and other psychiatric conditions [13]. Accordingly, we adopted a broad, dimensional conceptualisation of psychological distress rather than restricting inclusion to formally diagnosed psychiatric conditions. This approach was intended to capture the full range of volunteers’ emotionally distressing experiences that may not meet diagnostic thresholds. Quantitative studies were eligible if they assessed psychological burden-related outcomes or psychological disorders (e.g., depressive symptoms, affective disorders, anxiety-related disorders, burnout, or related constructs) using clearly described outcome measures. Qualitative studies were eligible if their research question specifically addressed emotional or psychological distress, burden, or strain, or if they reported volunteers’ experiences of these phenomena. These experiences ranged from general accounts of stress, burden, or strain in participant quotations or authors’ interpretations (e.g., “I feel burdened by…”, “X stresses me”, “I cannot cope with X”) to descriptions consistent with specific psychological conditions (e.g., “I feel depressed because of…”, “X makes me anxious”), provided that they were explicitly linked to factors associated with these experiences. A formal clinical diagnosis or diagnostic labelling was not required for inclusion. Eligibility criteria are shown in Table 1. Quantitative and qualitative study designs as well as mixed-methods studies were considered for analysis. Unpublished studies (i.e., conference abstracts, dissertations) were included if they met the study design criteria. Case reports, case series, and reviews were excluded. Studies focusing on mixed populations (e.g., volunteers and healthcare professionals combined) were included only if results for the subpopulation of end-of-life care volunteers were reported separately.

**Table 1:** Eligibility criteria

Inclusion criteria	Exclusion criteria
Studies reporting on factors associated with psychological distress	Studies focusing exclusively on paid healthcare staff, such as nurses or caregivers, rather than volunteers
Studies reporting on volunteers engaged in providing care or support in end-of-life care settings, including hospice volunteers, palliative care volunteers (both institutional and home-based), volunteers assisting with terminally ill patients in any palliative or end-of-life care environment	Studies involving participants in non-care roles only (e.g. volunteers working in administrative roles or fundraising)
All age groups (volunteers and care recipients)	Studies that focus on volunteers in non-end-of-life care settings (e.g. general hospital volunteers, community volunteers without palliative care tasks)
Original quantitative and qualitative studies (including mixed-methods studies)	Case reports, case series, and reviews
Studies from all geographical regions	

### Search strategy and study selection process

A systematic search *of* five electronic databases (MEDLINE, EMBASE, PsycINFO, Cochrane Database and Web of Science) was conducted in November 2024. The search strategy combined title/abstract keywords and Medical Subject Headings (MeSH) covering the population (volunteers), the context (end-of-life care) and the phenomenon of interest (psychological distress) (see Data Supplement). No restrictions regarding language or publication date were applied. In addition, citation tracking and reference list screening of all included studies were performed to identify additional relevant publications. All titles and abstracts were screened independently by two reviewers (LM and AP), a hospice volunteer and a palliative care physician with expertise in quantitative and qualitative research. In cases where relevance was unclear from the title or abstract, the full text was retrieved and assessed. Cases of uncertainty in study selection were resolved through discussion and consensus, which was necessary for two publications.

### Data collection process

For each included study, detailed information was extracted using a standardised data form including first author, year of publication, country of origin, population characteristics, sample size, factors studied, statistical analysis, results, and quality score. If needed, references that were not written in English, German, French or Spanish were translated using an online translation tool (Google Translate [14]). No specific systematic review management software was used.

### Quality assessment and strength of evidence

Double quality assessment was performed independently by LM and AP using the validated Standard Quality Assessment Criteria for Evaluating Primary Research Papers from a Variety of Fields (QualSyst) tool [15]. QualSyst comprises distinct yet complementary checklists for quantitative and qualitative studies and is therefore well suited to systematic reviews that include both forms of evidence. It provides structured criteria for appraising methodological quality across heterogeneous study designs. Details of the appraisal domains included in QualSyst are reported in the Supplementary Data. In accordance with previously established methodologies [16, 17], records providing quantitative evidence were classified into three categories of quality based on their assigned summary outcome QualSyst scores to prepare for the analysis stage: ‘low quality’ for scores below 50%, ‘medium quality’ for scores ranging from 50% to 70%, and ‘high quality’ for scores of 70% or higher.

### Synthesis of results

We initially planned to conduct a mixed-methods systematic review, integrating quantitative and qualitative evidence using a convergent synthesis approach. However, during full-test assessment and data extraction, substantial heterogeneity precluded meaningful integration of quantitative and qualitative findings. Consequently, we adopted a parallel synthesis approach, reporting quantitative and qualitative separately while maintaining a common review question.

### Quantitative data

**Fig. 1:**
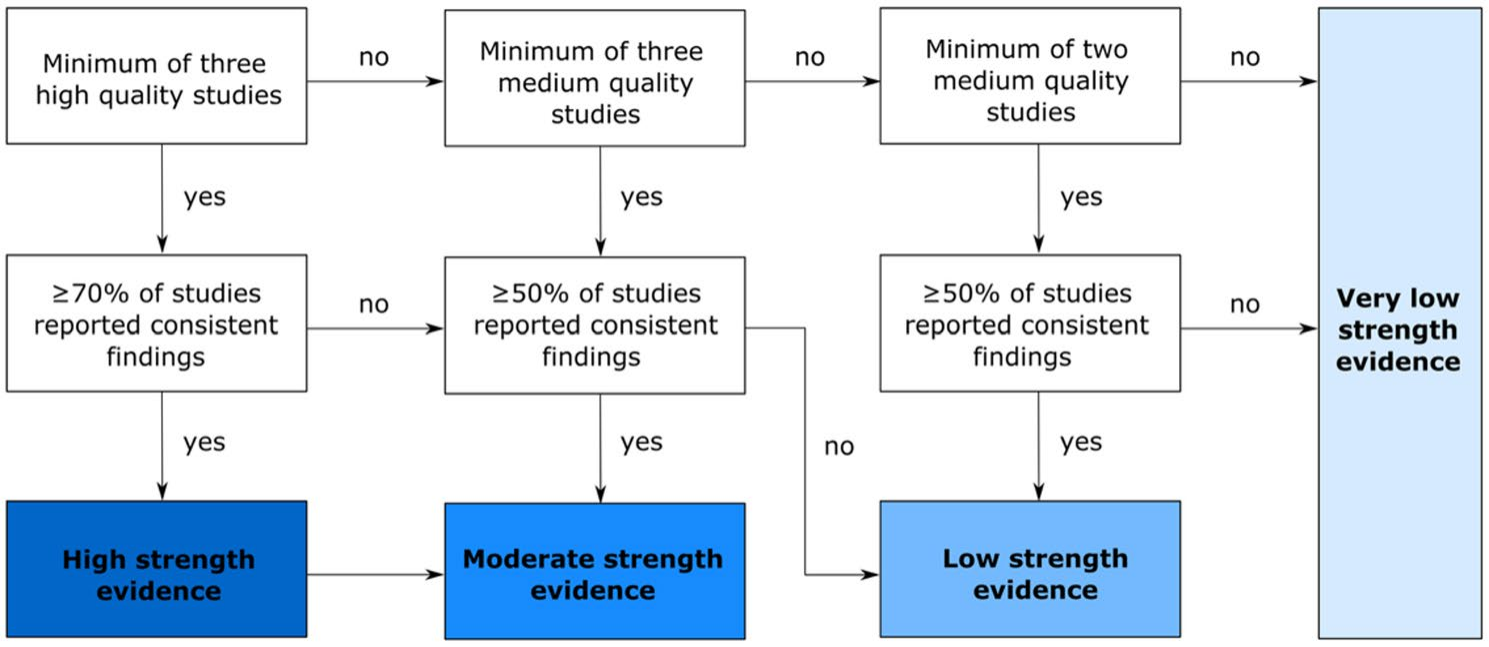
Algorithm for grading the strength of evidence based on Bone et al. adapted from Gomes and Higginson [17, 18]. Quality of individual studies was assessed by the Qualsyst tool [15]

For the quantitative studies, relative measures of associations (e.g., relative risk, odds ratios, or hazard ratios) or differences in means for continuous variables, accompanied by their 95% confidence intervals and significance levels, were extracted. A positive association between factors and the dependent variables was reported if there was a statistically significant increased likelihood of psychological distress (*p* < 0.05), a negative association if there was a statistically significant decreased likelihood of psychological distress (*p* < 0.05), and no association if there was no statistically significant effect (*p* ≥ 0.05). For each variable, the strength of the available evidence across all studies was assessed using an algorithm based on the framework established by Bone et al. adapted from Gomes and Higginson [17] (see Fig. 1). The overall strength of evidence for each factor was evaluated by considering the number of studies, their QualSyst scores, and the consistency of findings across the studies. In instances where an equal number of studies indicated opposing directions of association, the relationship was classified as inconclusive. For an overview of the algorithm see Fig. 1. Given the heterogeneity of study populations, methodological variations, and differences in summary outcome measures, the pooling of results through meta-analysis was not feasible. Therefore, the findings are presented narratively.

### Qualitative data

For qualitative data, we conducted a thematic synthesis informed by the approach described by Thomas and Harden [19]. This involved line-by-line coding of the results sections of included studies, followed by the organisation of codes into related categories to develop descriptive themes. LM and AP independently coded each included record using MAXQDA [20]. New codes were generated inductively when novel factors related to psychological distress or protection were identified, and these codes were subsequently organised into a hierarchical coding structure.

After coding all included studies, both authors jointly reviewed the complete code system and agreed on a consensus-based code tree. Any discrepancies in coding were resolved through discussion.

In the final stage, higher-order analytical themes were generated by iteratively comparing categories and further interpreting the descriptive themes to move beyond the content of the primary studies.

## Results

### Selection of relevant studies

**Fig. 2:**
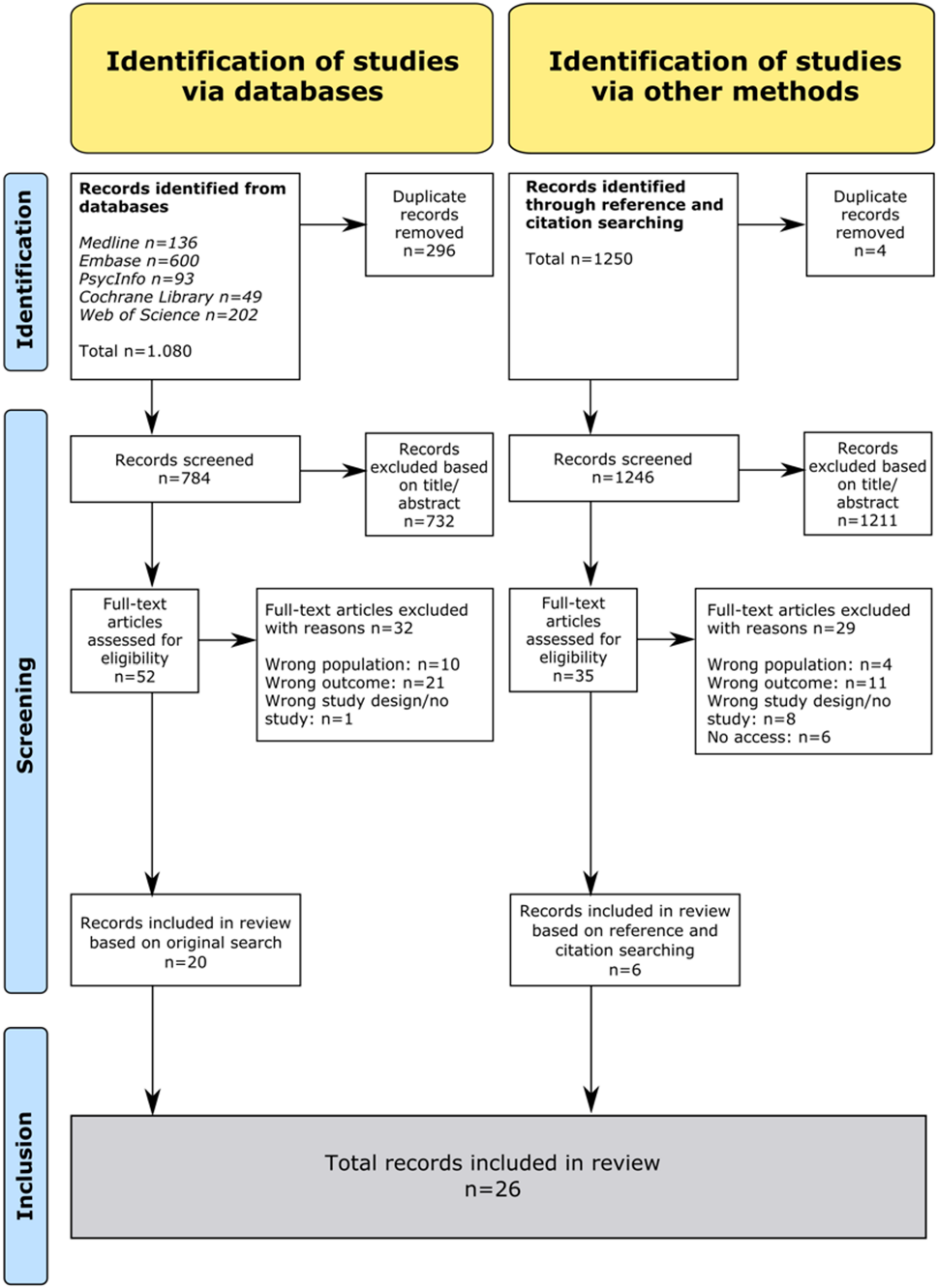
PRISMA Flowchart

A total of 1,080 records were identified through database searching, and an additional 1,250 records were identified through reference list searches. After removing 305 duplicates and screening 2,025 titles and abstracts, the full texts of 87 articles were assessed for eligibility. Finally, 26 studies met the inclusion criteria. The selection process is displayed in the PRISMA flowchart in Fig. 2.

### Study characteristics

We included 26 publications involving more than 2,000 participants, of which 20 reported quantitative data [21–40] and six were qualitative studies [41–46]. All articles were written in English, except for one in Croatian and one in German. 15 studies originated in North America (Canada, USA), five in Asia (China, Hong Kong, South Korea) and five in Europe (Croatia, Germany, Hungary, Netherlands, UK) and one in Australia. 23 studies were published as scientific articles, three as dissertations. The publishing journals primarily focused on palliative care and hospice care (*n* = 14), psychology (*n* = 3), thanatology (*n* = 3), and other disciplines (*n* = 3). The included studies spanned nearly four decades (1984–2023), with over half (*n* = 14) published after 2011.

### Quality assessment and strength of evidence

Of the 20 studies providing quantitative evidence thirteen were rated as high quality (≥ 70%) and seven as medium quality (50–69%), according to QualSyst scores. None of the studies were based on sample size calculations and most studies did not control for confounding. Additionally, estimates of variance were underreported. Furthermore, the six included qualitative studies had notable methodological weaknesses, particularly limited reporting of sampling strategies (mostly partial/absent), inconsistent use of credibility checks (often not reported), and only partial reflexivity across all studies. Reporting of data collection and especially data analysis also varied, with some studies providing only partial or insufficient detail. Detailed results of the quality appraisal are reported in the Supplementary Material.

### Synthesis of results

To facilitate reporting, the investigated factors were categorised into patient-related factors, volunteer-related factors, service-related factors, factors concerning volunteer–patient interaction, factors concerning volunteer–family interaction, and stress symptoms. These categories were developed inductively during data synthesis. Based on the specific outcomes assessed in the included studies, quantitative results were grouped into the following categories of psychological distress:

(i) anxiety;
(ii) death anxiety;
(iii) depression;
(iv) burnout;
(v) perceived stress.

### Quantitative results

For clarity and reporting consistency, only quantitative findings supported by at least a moderate level of evidence are presented below. Table 2 provides an overview of all identified factors and their associations with psychological distress. Importantly, no factors demonstrated strong evidence of association with any specific type of psychological distress. Findings rated as low or very low certainty are reported in the Supplementary Materials.

### Death anxiety

There was moderate evidence that age was not associated with increased levels of death anxiety. Similarly, moderate evidence indicated that volunteer training, as well as volunteering experience or duration, was not associated with higher levels of death anxiety. Additional moderate evidence suggested that better health and well-being were negatively associated with death anxiety.

### Depression

Regarding depression, we identified moderate evidence that volunteer training was negatively associated with depression scores.

**Table 2:** Overview of identified factors and evidence of their associations with psychological distress, restricted to results with at least moderate strength of evidence

Factor	Association	First author	Year of publication	Number of stars indicating quality	Overall Association	Strength of evidence
Death Anxiety						
Volunteer-related factors						
Age	=	Zana	2020	***	⇒	moderate
	=	Gillaspie	1993	***		
	=	Barrick	1985	**		
	-	Keith	1997	**		
Health/Well-being	-	Lin	2025	***	⇓	moderate
	-	Zana	2020	***		
	=	Barrick	1985	**		
Service-related factors						
Volunteer training	=	Lin	2025	***	⇒	moderate
	=	Lee	2019	***		
	=	Claxton-Oldfield	2007	**		
	=	Keith	1997	**		
	=	Barrick	1985	**		
	-	Woo	2018	***		
	-	Scherwitz	2006	***		
Experience/time trend	=	Keith	1997	**	⇒	moderate
	=	Barrick	1985	**		
	=	Robbins	1992	***		
	-	Nissim	2014	***		
	-	Lin	2025	***		
Depression						
Service-related factors						
Volunteer training	-	Huynh	2011	***	⇓	moderate
	-	Scherwitz	2006	***		
	=	Keith	1997	**		

Direction of association found in individual studies indicated as − (significant negative association), = (no significant association). Number of stars indicating quality: with *** = high quality (⩾ 70% QualSyst), ** = medium quality (50–69% QualSyst). Overall association is indicated as ⇒ (overall evidence for no significant association), ⇓ (overall evidence for negative association)

### Qualitative results

Through our qualitative analysis, we pinpointed triggering and guarding elements for psychological distress, categorised into six analytical themes: Exposure to suffering and existential vulnerability, Emotional proximity and boundary management, Negotiating death attitudes and moral dilemmas, Drawing on individual resources to manage emotional demands, Establishing one’s role within the healthcare system, and Implications for volunteer health.

### Exposure to suffering and existential vulnerability

Witnessing patient and family suffering, as well as existential vulnerability manifested in unexpected deterioration and physical disfigurement, contributed to psychological burden among end-of-life care volunteers [44, 46].

*“When I went to visit her there*,* I was ever so shocked at how she looked. […] When I came out*,* I was absolutely shaking from head to foot. I was not sure what I was expecting. I hadn’t seen anybody so yellow and dying.” (participant quotation)* [44]

*“…it was terrifying for me because it was the first time I had to tell a child who had openly asked me what was happening to his mother” (participant quotation)* [46]

### Emotional proximity and boundary management

Volunteers’ psychological burden appeared to arise from the tension between emotional proximity to patients and deliberate boundary management. Distress was heightened when volunteers supported younger patients, identified closely with patients, or accompanied the same patient over a prolonged period, particularly when a personal relationship developed [41, 44, 46]. Volunteers described self-care strategies aimed at preserving professional distance (e.g., limiting time commitments or undertaking less patient-facing tasks) [41] and at preventing psychological harm through restorative breaks from volunteering [41, 44].

*“You do develop a very close deep relationship with the patient and then there is real grief when the patient passes away.” (participant quotation)* [41]

*‘There have been one or two cases where the volunteers have said that they may need to rest it for a while. People say ‘I enjoy it but I just I need a break’. (participant quotation)* [44]

### Negotiating death attitudes and moral dilemmas

Volunteers’ psychological responses were shaped by their attitudes toward death and their perceived moral agency within care situations. Viewing death as a natural process or as a merciful release appeared to provide a framework that mitigated psychological burden [43]. In contrast, experiences of moral ambivalence such as feeling prevented from delivering appropriate care, encountering patients who refused help despite evident need, or engaging in discussions about euthanasia generated emotional strain [41, 45]. These situations seemed to create internal tension and uncertainty for volunteers.

*“It is a bit sad*,* but by the time*,* these things happen*,* you also feel that that person is out of their misery.” (participant quotation)* [44]

*“Especially if these wishes go against what the caregiver considers good care*,* this can lead to moral challenges between respecting the patient’s autonomy and promoting what is seen as best for the patient.” (authors’ interpretation)* [42]

### Drawing on individual resources to manage emotional demands

Individual resources appeared to strengthen volunteers’ capacity to cope with the psychological demands of end-of-life care. Life experience and the knowledge associated with older age were described as facilitating resilience in emotionally challenging situations [44]. Similarly, faith provided a framework for meaning-making, helping volunteers make sense of their experiences and mitigate psychological distress [44].

*I know that my God keeps me going. But there have been times*,* when I thought that I couldn’t cope with it*,* but then I thought that if we are all going to be here forever the world would fall apart. (participant quotation)* [44]

Perceived expertise in end-of-life care developed through practical experience and formal volunteer training also functioned as an important psychological resource [44, 45]. In contrast, a perceived lack of expertise including inadequate training was identified as a source of distress [42, 43]. Communication challenges such as interacting with poorly informed patients and breaking bad news were described as significant sources of psychological burden [45].

*“… it’s made me less afraid to die. Less afraid of my own deathbed experience. And that’s not a small thing because I was raised by a grandmother who’s deeply religious and she was very terrified of her own death.” (participant quotation)* [45]

*“One woman noted that she did not feel the training helped her deal with the patient’s death. She felt “unprepared” and did not know what to do.” (authors’ interpretation)* [43]

### Establishing one’s role within the care system

End-of-life care volunteers emphasised their distinct position within the healthcare system, describing the need to adapt to varying circumstances while asserting their role and function. The perceived suitability and clarity of the volunteer role acted as a protective factor against psychological distress [43, 44]. In contrast, personal sacrifices related to irregular working hours and the unpredictable nature of the work were reported emotionally burdensome [46].

*“So*,* they call me while I’m at work*,* they call me at night while I’m seeing someone else*,* they call me early in the morning*,* so that’s actually a problem. I’m available 24/7…” (participant quotation)* [46]

Furthermore, team dynamics played a critical role in shaping psychological strain. Supportive team environments, particularly those characterised by peer support, were described as protective [43, 46]. Conversely, a lack of teamwork, poor cooperation, and interpersonal conflict within teams were associated with heightened psychological burden [43, 46]. In particular, uncertainty about role responsibilities and role boundaries was associated with internal psychological tension among end-of-life care volunteers. Reported stressors included role ambiguity in supporting patients and families [42–44, 46], and perceived status ambiguity within the interdisciplinary end-of-life care team [42–44].

*“Everyone has their own path*,* their own program. There is not much joint cooperation and teamwork*,* so that could be a source of stress in terms of demotivation for work.” (participant quotation)* [46]

*“Regardless of organizational type*,* 35%* [6] *of the volunteers interviewed noted that they were not always clear about their role in the organization and*,* as a result*,* felt job stress. Should they attend staff meetings? Do they have free access to their patient’s chart? Are they expected to spend time with other staff discussing the patient? Is their role as a patient or staff advocate?” (authors’ interpretation)* [43]

Additionally, volunteers described recognition by patients as an important factor influencing their psychological well-being. When their efforts were perceived as unacknowledged, whether through a lack of appreciation, rejection by patients, feelings that their time was wasted, or the absence of a shared understanding of the goals of their involvement, this contributed to psychological strain [42, 43, 46].

*“If someone invites you to their house to help them with advice or something*,* then you come to the house*,* get to know each other*,* have small talks and then you get rejected and then you kill yourself wondering why they even invited us and what the point of us coming to the house is.” (participant quotation)* [46]

Furthermore, structural conditions shaped volunteers’ experiences. Rigid institutional protocols and regulations, alongside limited financial support for volunteer work, were described as constraining and as creating tensions around role expectations [41, 46]. Liability concerns further restricted volunteers from performing certain tasks (e.g., feeding), which limited their practical involvement and was perceived as undermining their usefulness to patients and families [41].

*“Both nurses and volunteers indicated that*,* in certain situations*,* protocols and regulations presented them with moral challenges*,* as they felt they were obstructing good care in some way.” (authors’ interpretation)* [42]

*“What is a problem for me and what makes me extremely sad is when I have to sit here and solve some bills or write some project for money.” (participant quotation)* [46]

### Implications for volunteer health

According to Bosnjak et al., end-of-life care volunteers reported implications for their own health. Psycholgical stress manifested in physical symptoms such as vomiting, headaches and sweating, as well as psychological symptoms such as difficulty concentrating, verbal aggression and nervousness [46].

*“Physical symptoms are headache…a heaviness in the head after the death of a person who was very dear to me…” (participant quotation)* [46]

## Discussion

By integrating quantitative and qualitative evidence, this review synthesises manifold factors associated with psychological distress in end-of-life care volunteers, while also highlighting substantial gaps in the current literature.

Our analysis of quantitative studies found some moderate evidence on factors associated with death anxiety and depression scores. Death anxiety and depression are well-established mental health concerns, not only within the general populace [47, 48], but also within the healthcare community [49, 50]. Psychological distress exerts statistically significant negative effects on health outcomes [51], while at the societal level it represents a cost driver in healthcare systems [52, 53]. Although our review design does not permit causal inferences, it is plausible that psychological distress — particularly death anxiety identified in this review — may increase vulnerability to poorer health and reduced well-being among end-of-life care volunteers. These findings underscore the importance of understanding underlying mechanisms and contextual factors that may exacerbate or mitigate psychological burden in volunteers working in end-of-life care.

In line with previous research on hospice staff [54], which found no consistent associations between staff characteristics and well-being, we likewise did not identify strong evidence linking volunteers’ sociodemographic factors to psychological distress. However, as only a few studies addressed such variables, a broader evidence base is needed before such associations can be conclusively dismissed. Our research further supports existing evidence on burnout in hospice staff that did not find any associations with length of experience [55]. However, more research is needed to identify vulnerable subgroups of end-of-life care volunteers to enable targeted supports that promote volunteer well-being and reduce psychological burden and related attrition [56].

Consistent with evidence that increased training is associated with improved psychological well-being among hospice staff [54], our quantitative synthesis provided moderate evidence that volunteer training may be protective against depression. Educational interventions in particular have shown promise in fostering resilience within the healthcare workforce [57]. Although the overall body of research on volunteer training remains limited, existing studies suggest that such training can improve knowledge and confidence across various domains of palliative care [58], and may also enhance volunteers’ involvement in the organisation of care [3]. Still, there is a lack of evidence on how best to train and support volunteers while ensuring high standards of care for palliative care patients and their families [59]. In our review, considerable heterogeneity in training designs, including content, duration, timing, and delivery formats, may also have limited comparability across studies. This variability underscores the importance of identifying which specific training components provide the greatest benefit for volunteers. Clarifying these elements would enable the development of more targeted and efficient curricula that may help to reduce psychological distress in end-of-life care volunteerism.

Included qualitative studies typically described volunteer burden in broader terms such as general psychological distress, emotional strain, or overwhelm as opposed to standardised, clinically anchored constructs such as death anxiety and depression. With only six qualitative studies included and some exhibiting important methodological limitations the evidence base is limited and the findings should be interpreted cautiously. However, interestingly, these studies shed light on patient-related, service-related, and interactional factors that appear highly relevant for volunteers’ mental health, but in turn have scarcely been addressed in quantitative research. Existing quantitative studies have focused predominantly on volunteer-level factors, leaving relational and systemic influences underexplored. Paradoxically, the very aspects that may distinguish end-of-life care volunteerism — such as close engagement with vulnerable patients, families, and interdisciplinary teams — are those least examined in quantitative research. As a result, what makes end-of-life volunteering unique may remain underexplored, underscoring the need for future studies to systematically investigate service-related and relational dimensions. Such work could help clarify whether the drivers of psychological distress and resilience in end-of-life care are truly distinct or instead reflect broader challenges of volunteerism.

## Limitations

This review has several important limitations. While the inclusion of both qualitative and quantitative studies provided valuable insights, substantial heterogeneity in study designs, populations, and outcome measures precluded a meta-analysis. The authors’ conceptual understanding of psychological distress likely influenced study selection and interpretation, and may not fully align with alternative definitions of these constructs. As such, different operationalisations of these concepts could have resulted in different inclusion decisions and interpretations of the findings. The quantitative studies further allowed the identification of associations but not causal relationships. The overall evidence base was relatively limited, with few studies specifically addressing volunteer distress in end-of-life care. Most available research originated from high-income countries, constraining the generalisability of findings to other settings. In addition, some included studies dated back to the 1980s, which may limit the relevance of their findings to contemporary healthcare contexts given changes in professional roles, training, and cultural expectations. Considerable variation in the scope and nature of volunteer roles across settings also made direct comparisons difficult. Finally, while a rigorous search strategy was applied, it is possible that relevant studies were missed including some identified in an earlier review of end-of-life care volunteers’ experiences [10], which may have affected the completeness of the evidence base.

## Conclusion

This review highlights a fragmented and limited evidence base on psychological distress among end-of-life care volunteers. Quantitative evidence suggests that training appears to offer some protection against depression, but variations in programme content and delivery limit firm conclusions. Although qualitative evidence is scarce, it foregrounds potential patient-, interaction- and service-level factors that are rarely examined quantitatively. Future studies should employ rigorous, theory-driven, and longitudinal designs to elucidate the mechanisms underlying distress and resilience in this vital but under-researched group of care providers.

## Supplementary Information

Supplementary Material 1.

## Data Availability

The data generated in this study are available from the corresponding author upon request.

## References

[1] Fink M, Schultz O. Das Ehrenamt in der Sterbebegleitung: gegenwärtige Herausforderungen und künftige Chancen. Bielefeld: transcript; 2021.

[2] Abel EK. The hospice movement: Institutionalizing innovation. Int J Health Serv. 1986;16(1):71–85. 3514497 10.2190/RQBV-J2PG-VFNM-1H97

[3] Vanderstichelen S, Houttekier D, Cohen J, Van Wesemael Y, Deliens L, Chambaere K. Palliative care volunteerism across the healthcare system: a survey study. Palliat Med. 2018;32(7):1233–45. 29737245 10.1177/0269216318772263 PMC6050945

[4] Candy B, France R, Low J, Sampson L. Does involving volunteers in the provision of palliative care make a difference to patient and family wellbeing? A systematic review of quantitative and qualitative evidence. Int J Nurs Stud. 2015;52(3):756–68. 25205665 10.1016/j.ijnurstu.2014.08.007

[5] Jenkinson CE, Dickens AP, Jones K, Thompson-Coon J, Taylor RS, Rogers M, et al. Is volunteering a public health intervention? A systematic review and meta-analysis of the health and survival of volunteers. BMC Public Health. 2013;13(1):773. 23968220 10.1186/1471-2458-13-773 PMC3766013

[6] Cherny N, Werman B, Kearney M. Burnout, compassion fatigue, and moral distress in palliative care. In: Levan N, editor. Oxford Textbook of Medicine. Oxford: Oxford University Press; 2015;9:91–122.

[7] Baqeas MH, Davis J, Copnell B. Compassion fatigue and compassion satisfaction among palliative care health providers: a scoping review. BMC Palliat care. 2021;20(1):88. 34162388 10.1186/s12904-021-00784-5 PMC8220432

[8] Pawłowski L, Lichodziejewska-Niemierko M, Pawłowska I, Leppert W, Mróz P. Nationwide survey on volunteers’ training in hospice and palliative care in Poland. BMJ Supportive Palliat Care. 2019;9(3):e25–e. 10.1136/bmjspcare-2015-000984 27474087

[9] Claxton-Oldfield S. Hospice palliative care volunteers: The benefits for patients, family caregivers, and the volunteers. Palliat Support Care. 2015;13(3):809–13. 24901841 10.1017/S1478951514000674

[10] Coleman H, Walshe C. What are the emotional experiences of being a volunteer in palliative and end-of-life care settings? A systematic review and thematic synthesis. J Pain Symptom Manag. 2021;62(3):e232–47. 10.1016/j.jpainsymman.2021.02.025 33647423

[11] Page MJ, McKenzie JE, Bossuyt PM, Boutron I, Hoffmann TC, Mulrow CD, et al. The PRISMA 2020 statement: an updated guideline for reporting systematic reviews. BMJ. 2021;372. 10.1136/bmj.n71 PMC8005924 33782057

[12] Tong A, Flemming K, McInnes E, Oliver S, Craig J. Enhancing transparency in reporting the synthesis of qualitative research: ENTREQ. BMC Med Res Methodol. 2012;12(1):181. 23185978 10.1186/1471-2288-12-181 PMC3552766

[13] American Psychology Association. APA Dictionary of Psychology: psychological distress Washington, USA; 2018. Available from: https://dictionary.apa.org/psychological-distress. Updated 04/19/201805/28/2026.

[14] Google. Google Translate. 2025.

[15] Kmet LM, Cook LS, Lee RC. Standard quality assessment criteria for evaluating primary research papers from a variety of fields. 2004.

[16] Zehnder AR, Pedrosa Carrasco AJ, Etkind SN. Factors associated with hospitalisations of patients with chronic heart failure approaching the end of life: A systematic review. Palliat Med. 2022;36(10):1452–68. 36172637 10.1177/02692163221123422 PMC9749018

[17] Bone AE, Evans CJ, Etkind SN, Sleeman KE, Gomes B, Aldridge M, et al. Factors associated with older people’s emergency department attendance towards the end of life: a systematic review. Eur J Pub Health. 2019;29(1):67–74. 30481305 10.1093/eurpub/cky241 PMC6345149

[18] Gomes B, Higginson IJ. Factors influencing death at home in terminally ill patients with cancer: systematic review. BMJ. 2006;332(7540):515–21. 16467346 10.1136/bmj.38740.614954.55 PMC1388126

[19] Thomas J, Harden A. Methods for the thematic synthesis of qualitative research in systematic reviews. BMC Med Res Methodol. 2008;8(1):45. 18616818 10.1186/1471-2288-8-45 PMC2478656

[20] Software V. MAXQDA24. 2024.

[21] Zana Á, Kegye A, Czeglédi E, Hegedűs K. Differences in well-being and fear of death among female hospice employees and volunteers in Hungary. BMC Palliat care. 2020;19(1):58. 32331526 10.1186/s12904-020-00550-z PMC7183127

[22] Woo HY, Yeun YR. Impact of a palliative care education program on Korean hospice volunteers: motivation, death anxiety, and communication with the dying. Korean J Hospice Palliat Care. 2018;21(2):58–64.

[23] Wang Q, Chan IK, Lou VW. Effectiveness of a holistic capacity-building program for volunteers in community-based end-of-life care. Res Social Work Pract. 2020;30(4):408–21.

[24] Wach D, Hentschel L, Rosenkranz B, Rudolf M. Motivationaler und gesundheitsschädigender Prozess bei Deutschen Hospizhelfern. Zeitschrift für Arbeits- und Organisationspsychologie. 2017;61(3):137–51.

[25] Scherwitz L, Pullman M, McHenry P, Gao B, Ostaseski F. A contemplative care approach to training and supporting hospice volunteers: a prospective study of spiritual practice, well-being, and fear of death. Explore. 2006;2(4):304–13. 16846818 10.1016/j.explore.2006.04.001

[26] Robbins RA. Death competency: a study of hospice volunteers. Death Stud. 1992;16(6):557–69. 10122686 10.1080/07481189208252598

[27] Robbins RA. Death anxiety, death competency and self-actualization in hospice volunteers. Hospice J. 1991;7(4):29–35. 10.1080/0742-969x.1991.11882708 1809664

[28] Paradis LF, Usui WM. Hospice volunteers: The impact of personality characteristics on retention and job performance. Hospice J. 1987;3(1):3–30. 10.1080/0742-969x.1987.11882578 3646166

[29] Nissim R, Emmerson D, O’Neill B, Marchington K, Draper H, Rodin G. Motivations, satisfaction, and fears of death and dying in residential hospice volunteers: A prospective longitudinal study. Am J Hospice Palliat Medicine^®^. 2016;33(4):335–9. 10.1177/1049909114559830 25425739

[30] Lin Z, Lou VW, Chan WCH. Validating the self-competence in death work scale for end-of-life care volunteers. BMC Palliat Care. 2025;24(1):35. 39905427 10.1186/s12904-025-01666-w PMC11792580

[31] Lee J, Lee J-E. A palliative care program for volunteers in a community setting: a mixed-methods pilot study. Am J Hospice Palliat Medicine^®^. 2020;37(6):455–64. 10.1177/1049909119895213 31859524

[32] Keith PM. Investigation of a typology of life and death among hospice workers. Sociol Spectr. 1997;17(4):417–35.

[33] Jo M, Na H, Jung Y-E. Mediation effects of compassion satisfaction and compassion fatigue in the relationships between resilience and anxiety or depression among hospice volunteers. J Hospice Palliat Nurs. 2020;22(3):246–53. 10.1097/NJH.0000000000000640 32168086

[34] Huynh J-Y, Winefield AH, Xanthopoulou D, Metzer JC. Burnout and connectedness in the job demands–resources model: Studying palliative care volunteers and their families. Am J Hospice Palliat Medicine^®^. 2012;29(6):462–75. 10.1177/1049909111430224 22207713

[35] Hayslip B Jr, Sethi A, Pinson MW, Carpenter C. Predicting attrition among hospice volunteers. OMEGA-Journal Death Dying. 2021;84(1):289–306. 10.1177/0030222819889830 31775573

[36] Halliday L. Burnout and job satisfaction among hospice volunteers. Long Beach: California State University; 1997.

[37] Gillaspie MH. Using the threat index to predict death anxiety, sense of purpose, and effectiveness of hospice volunteer personnel. University of Florida; 1993.

[38] Claxton-Oldfield S, Crain M, Claxton-Oldfield J. Death anxiety and death competency: the impact of a palliative care volunteer training program. Am J Hospice Palliat Medicine^®^. 2007;23(6):464–8. 10.1177/1049909106294882 17211000

[39] Barrick DE. The effects of hospice volunteer training on death anxiety levels of hospice volunteer candidates. North Carolina State University; 1985.

[40] Amenta MM. Death anxiety, purpose in life and duration of service in hospice volunteers. Psychol Rep. 1984;54(3):979–84. 6473612 10.2466/pr0.1984.54.3.979

[41] Weeks LE, MacQuarrie C. Supporting the volunteer career of male hospice—Palliative care volunteers. Am J Hospice Palliat Medicine^®^. 2011;28(5):342–9. 10.1177/1049909110389322 21087948

[42] van den Bosch G, van Schaik M, Pasman HR, Janssens R, Widdershoven G, Metselaar S. Moral challenges of nurses and volunteers in dutch palliative care. A qualitative study. J Palliat Care. 2023;38(3):364–71. 35612868 10.1177/08258597221098129 PMC10350729

[43] Paradis LF, Miller B, Runnion VM. Volunteer stress and burnout: Issues for administrators. Hospice J. 1987;3(2–3):165–83. 10.1080/0742-969x.1987.11882598 3692450

[44] Dein S, Abbas SQ. The stresses of volunteering in a hospice: a qualitative study. Palliat Med. 2005;19(1):58–64. 15690869 10.1191/0269216305pm969oa

[45] Claxton-Oldfield S, Yoon H. Deathbed visions: Hospice palliative care volunteers’ experiences, perspectives, and responses. OMEGA-Journal Death Dying. 2025;91(3):1642–57. 10.1177/00302228231161815 36880116

[46] Bošnjak M, Rusac S. Izvori Stresa Kod Volontera u Palijativnoj Skrbi. Socijalna Psihijatrija. 2012;40:37–44.

[47] Bowling A, Iliffe S, Kessel A, Higginson IJ. Fear of dying in an ethnically diverse society: cross-sectional studies of people aged 65 + in Britain. Postgrad Med J. 2010;86(1014):197–202. 20354041 10.1136/pgmj.2009.084020 PMC2921269

[48] Necho M, Tsehay M, Birkie M, Biset G, Tadesse E. Prevalence of anxiety, depression, and psychological distress among the general population during the COVID-19 pandemic: A systematic review and meta-analysis. Int J Soc Psychiatry. 2021;67(7):892–906. 33794717 10.1177/00207640211003121

[49] Nia HS, Lehto RH, Ebadi A, Peyrovi H. Death anxiety among nurses and health care professionals: A review article. Int J community based Nurs midwifery. 2016;4(1):2. 26793726 PMC4709813

[50] Weibelzahl S, Reiter J, Duden G. Depression and anxiety in healthcare professionals during the COVID-19 pandemic. Epidemiol Infect. 2021;149:e46. 33557984 10.1017/S0950268821000303 PMC7900665

[51] Barry V, Stout ME, Lynch ME, Mattis S, Tran DQ, Antun A, et al. The effect of psychological distress on health outcomes: A systematic review and meta-analysis of prospective studies. J Health Psychol. 2020;25(2):227–39. 30973027 10.1177/1359105319842931

[52] Konnopka A, König H. Economic burden of anxiety disorders: a systematic review and meta-analysis. PharmacoEconomics. 2020;38(1):25–37. 31646432 10.1007/s40273-019-00849-7

[53] König H, König H-H, Konnopka A. The excess costs of depression: a systematic review and meta-analysis. Epidemiol psychiatric Sci. 2020;29:e30. 10.1017/S2045796019000180 PMC8061284 30947759

[54] Papworth A, Ziegler L, Beresford B, Mukherjee S, Fraser L, Fisher V, et al. Psychological well-being of hospice staff: systematic review. BMJ Supportive Palliat Care. 2023;13(e3):e597–611. 10.1136/spcare-2022-004012 37098444

[55] Pavelková H, Bužgová R. Burnout among healthcare workers in hospice care. Cent Eur J Nurs Midwifery. 2015;6(1):218–23.

[56] Claxton-Oldfield S, Claxton-Oldfield J. Should I stay or should I go: A study of hospice palliative care volunteer satisfaction and retention. Am J Hospice Palliat Medicine^®^. 2012;29(7):525–30. 10.1177/1049909111432622 22241460

[57] Rogers D. Which educational interventions improve healthcare professionals’ resilience? Med Teach. 2016;38(12):1236–41. 27573430 10.1080/0142159X.2016.1210111

[58] Elias H, Kisembe E, Nyariki S, Kiplimo I, Amisi J, Boit J, et al. Impact of training on knowledge, confidence and attitude amongst community health volunteers in the provision of community-based palliative care in rural Kenya. BMC Palliat Care. 2024;23(1):97. 38605309 10.1186/s12904-024-01415-5 PMC11007868

[59] Horey D, Street AF, O'Connor M, Peters L, Lee SF. Training and supportive programs for palliative care volunteers in community settings. Cochrane Database Syst Rev. 2015;(7):CD009500. 10.1002/14651858.CD009500.pub2. 10.1002/14651858.CD009500.pub2 PMC8917830 26189823

