# Supplementary material for: Factors associated with psychological distress among end-of-life care volunteers: a systematic review of quantitative and qualitative evidence
Source: BMC Palliat Care. 2026 Jun 26;25:182. doi: 10.1186/s12904-026-02201-1 (PMC13307674; doi:10.1186/s12904-026-02201-1)
Supplement: Supplementary file 1 — Supplementary Material 1. [file 12904_2026_2201_MOESM1_ESM.docx]

# Search strategy

## **PsycInfo (EBSCO Host) (1933-2024)**

(TI (volunteer* OR voluntar* OR unpaid) OR AB (volunteer* OR voluntar* OR unpaid) OR DE "Volunteers")

AND

(TI (palliative OR hospice* OR EOL OR ("end of life" OR "end-of-life")) OR AB (palliative OR hospice* OR EOL OR ("end of life" OR "end-of-life")) OR DE "Palliative Care" OR DE "Hospice Care" OR DE “terminally ill patients”)

AND

(TI (distress OR depression OR anxiety OR "adjustment disorder") OR AB (distress OR depression OR anxiety OR "adjustment disorder") OR DE "Psychological stress" OR DE “distress” DE "Depression" OR DE "Anxiety" OR DE "Adjustment Disorders" OR DE “affective disorders” OR DE “anxiety disorders”)

**EMBASE**

(volunteer* OR voluntar* OR unpaid OR 'voluntary worker'/exp OR 'hospital volunteer'/exp)

AND

(palliative OR hospice* OR EOL OR "end of life" OR "end-of-life" OR 'palliative therapy'/exp OR 'palliative nursing'/exp OR 'hospice care'/exp OR 'hospice'/exp OR 'hospice patient'/exp OR 'hospice nursing'/exp OR 'terminal care'/exp)

AND

(distress OR depression OR anxiety OR "adjustment disorder" OR 'distress syndrome'/exp OR 'psychological distress'/exp OR 'depression'/exp OR 'anxiety'/exp OR 'anxiety disorder'/exp OR 'fear of death/exp OR 'adjustment disorder'/exp)

**Medline (Ovid)**

(volunteer* or voluntar* or unpaid).ti,ab.

(volunteers or hospital volunteers).mp.

1 or 2

(palliative or hospice* or EOL or end of life or end-of-life).ti,ab.

(palliative care or palliative medicine or hospice care or hospices or terminal care or (hospice and palliative care nursing)).mp.

4 or 5

(distress or depression or anxiety or adjustment disorder).ti,ab.

(psychological distress or depression or depressive disorder or adjustment disorders or anxiety or anxiety disorders).mp.

7 or 8

3 and 6 and 9

**Cochrane Database**

(volunteer OR voluntary OR unpaid) AND (palliative OR hospice OR EOL OR "end of life" OR "end-of-life") AND (distress OR depression OR anxiety OR "adjustment disorder") in Title Abstract Keyword OR (volunteers OR "hospital volunteers") AND ("palliative care" OR "palliative medicine" OR "hospice care" OR hospices OR "terminal care" OR "hospice and palliative care nursing") AND ("psychological distress" OR depression OR "depressive disorder" OR "adjustment disorders" OR anxiety OR "anxiety disorders") in Keyword

**Web of science**

((TS=(volunteer* OR voluntar* OR unpaid)) OR (TS=(volunteers OR "hospital volunteers")))

AND

((TS=(palliative OR hospice* OR EOL OR "end of life" OR "end-of-life")) OR (TS=("palliative care" OR "palliative medicine" OR "hospice care" OR hospices OR "terminal care" OR ("hospice" AND "palliative care nursing"))))

AND

((TS=(distress OR depression OR anxiety OR "adjustment disorder")) OR (TS=("psychological distress" OR depression OR "depressive disorder" OR "adjustment disorders" OR anxiety OR "anxiety disorders")))

# Anxiety

Table 1: Direction of association found in individual studies indicated as + (significant positive association), − (significant negative association), = (no significant association). Number of stars indicating quality: with *** = high quality (⩾ 70% QualSyst), **= moderate quality (50-69%). Overall association is indicated as ↑ (overall evidence for positive association), x (overall evidence for no significant association), ↓ (overall evidence for negative association).

| **Factor** | **Association** | **First author** | **Year of publication** | **Number of stars indicating quality** | **Overall Association** | **Strength of evidence** |
| --- | --- | --- | --- | --- | --- | --- |
| **Volunteer-related factors** | | | | | | |
| Compassion fatigue | + | Jo | 2020 | ******* | ↑ | very low |
| Compassion satisfaction | − | Jo | 2020 | ******* | ↓ | very low |
| Coping with Death | = | Paradis | 1987 | ****** | x | very low |
| Resilience | − | Jo | 2020 | ******* | ↓ | very low |
| Understanding the Dying Person and His Family | = | Paradis | 1987 | ****** | x | very low |
| Volunteer Job Performance | = | Paradis | 1987 | ****** | x | very low |
| **Service-related** | | | | | | |
| Volunteer training | − | Scherwitz | 2006 | *** | ↓ | very low |

# Death anxiety

Table 2: Direction of association found in individual studies indicated as + (significant positive association), − (significant negative association), = (no significant association). Number of stars indicating quality: with *** = high quality (⩾ 70% QualSyst), **= moderate quality (50-69%). Overall association is indicated as ↑ (overall evidence for positive association), x (overall evidence for no significant association), ↓ (overall evidence for negative association). ^a^Personal Orientation Inventory

| **Factor** | **Association** | **First author** | **Year of publication** | **Number of stars indicating quality** | **Overall Association** | **Strength of evidence** |
| --- | --- | --- | --- | --- | --- | --- |
| **Volunteer-related factors** | | | | | | |
| Age | = | Zana | 2020 | ******* | x | moderate |
|  | = | Gillaspie | 1993 | ** |  |  |
|  | = | Barrick | 1985 | ****** |  |  |
|  | − | Keith | 1997 | ****** |  |  |
| Health/Well-being | − | Lin | 2025 | ******* | ↓ | moderate |
|  | − | Zana | 2020 | *** |  |  |
|  | = | Barrick | 1985 | ****** |  |  |
| Depression | + | Zana | 2020 | ******* | ↑ | low |
|  | + | Keith | 1997 | ****** |  |  |
| Education | = | Keith | 1997 | ****** | x | low |
|  | = | Gillaspie | 1993 | ****** |  |  |
| Income | = | Keith | 1997 | ****** | x | low |
|  | = | Gillaspie | 1993 | ****** |  |  |
| Marital status | = | Gillaspie | 1993 | ****** | x | low |
|  | = | Barrick | 1985 | ****** |  |  |
| Social comparisons/ Self-regard* | = | Keith | 1997 | ****** | x | low |
|  | − | Robbins | 1991 | ****** |  |  |
| Acceptance of Aggression* | = | Robbins | 1991 | ****** | x | very low |
| Capacity for Intimate Contact* | = | Robbins | 1991 | ****** | x | very low |
| Compassion fatigue | + | Hayslip | 2021 | ******* | ↑ | very low |
| Compassion satisfaction | − | Hayslip | 2021 | ******* | ↓ | very low |
| Existenciality* | = | Robbins | 1991 | ****** | x | very low |
| Feeling Reactivity* | = | Robbins | 1991 | ****** | x | very low |
| Gender | = | Gillaspie | 1993 | ****** | x | very low |
| Inner-directed* | − | Robbins | 1991 | ****** | ↓ | very low |
| Life satisfaction | − | Keith | 1997 | ****** | ↓ | very low |
| Nature of Man, Constructive* | = | Robbins | 1991 | ****** | x | very low |
| Nursing background | = | Barrick | 1985 | ****** | x | very low |
| Perceived stress | + | Zana | 2020 | ******* | ↑ | very low |
| Previous/current experience with death/life-limiting illness | = | Barrick | 1985 | ****** | x | very low |
| Reasons for wanting to become a hospice volunteer | = | Barrick | 1985 | ****** | x | very low |
| Religiosity | − | Gillaspie | 1993 | ** | ↓ | very low |
| Religious orientation | = | Barrick | 1985 | ** | x | very low |
| Religious service attendance | = | Barrick | 1985 | ** | x | very low |
| Resilience | − | Hayslip | 2021 | ******* | ↓ | very low |
| Self-acceptance^a^ | − | Robbins | 1991 | ****** | ↓ | very low |
| Self-actualizing Value* | = | Robbins | 1991 | ****** | x | very low |
| Spontaneity* | = | Robbins | 1991 | ****** | x | very low |
| Synergy* | = | Robbins | 1991 | ****** | x | very low |
| Threat Index Score | + | Gillaspie | 1993 | ****** | ↑ | very low |
| Time Competence* | − | Robbins | 1991 | ****** | ↓ | very low |
| Vital exhaution | + | Zana | 2020 | ******* | ↑ | very low |
| Work orientation | = | Keith | 1997 | ****** | x | very low |
| **Service-related** | | | | | | |
| Experience/time trend | = | Keith | 1997 | ****** | x | moderate |
|  | = | Barrick | 1985 | ****** |  |  |
|  | = | Robbins | 1992 | ******* |  |  |
|  | − | Nissim | 2014 | ******* |  |  |
|  | − | Lin | 2025 | ******* |  |  |
| Volunteer training | = | Lin | 2025 | ******* | x | moderate |
|  | = | Lee | 2019 | ******* |  |  |
|  | = | Claxton-Oldfield | 2007 | ****** |  |  |
|  | = | Keith | 1997 | ****** |  |  |
|  | = | Barrick | 1985 | ****** |  |  |
|  | − | Woo | 2018 | ******* |  |  |
|  | − | Scherwitz | 2006 | ******* |  |  |
| No. hours per week in hospice work | = | Keith | 1997 | ****** | x | very low |
| Resident and family care volunteers | − | Nissim | 2014 | ******* | ↓ | very low |
| Withdrawal | + | Amenta | 1984 | ****** | ↑ | very low |

# Depression

Table 3: Direction of association found in individual studies indicated as + (significant positive association), − (significant negative association), = (no significant association). Number of stars indicating quality: with *** = high quality (⩾ 70% QualSyst), **= moderate quality (50-69%). Overall association is indicated as ↑ (overall evidence for positive association), x (overall evidence for no significant association), ↓ (overall evidence for negative association).

| **Factor** | **Association** | **First author** | **Year of publication** | **Number of stars indicating quality** | **Overall Association** | **Strength of evidence** |
| --- | --- | --- | --- | --- | --- | --- |
| **Volunteer-related factors** | | | | | | |
| Compassion fatigue | − | Hayslip | 2021 | ******* | ↓ | low |
|  | − | Jo | 2020 | ******* |  |  |
| Death-Anxiety | + | Zana | 2020 | ******* | ↑ | low |
|  | + | Keith | 1997 | ** |  |  |
| Resilience | − | Hayslip | 2021 | ******* | ↓ | low |
|  | − | Jo | 2020 | ******* |  |  |
| Age | = | Keith | 1997 | ** | x | very low |
| Compassion satisfaction | − | Hayslip | 2021 | ******* | ↓ | very low |
|  | = | Jo | 2020 | ******* |  |  |
| Cynicism | + | Huynh | 2011 | ******* | ↑ | very low |
| Education | = | Keith | 1997 | ** | x | very low |
| Exhaustion | + | Huynh | 2011 | ******* | ↑ | very low |
| Family Support | − | Huynh | 2011 | ******* | ↓ | very low |
| Income | = | Keith | 1997 | ** | x | very low |
| Life satisfaction | − | Keith | 1997 | ****** | ↓ | very low |
| Religiosity | − | Gillaspie | 1993 | ****** | ↓ | very low |
| Social comparisons/ Self-regard | + | Keith | 1997 | ****** | ↑ | very low |
| Work–home conflict | + | Huynh | 2011 | ******* | ↑ | very low |
| Work orientation | − | Keith | 1997 | ****** | ↓ | very low |
| **Service-related** | | | | | | |
| Volunteer training | − | Huynh | 2011 | *** | ↓ | moderate |
|  | − | Scherwitz | 2006 | *** |  |  |
|  | = | Keith | 1997 | ****** |  |  |
| Appreciation by Co-Workers | − | Huynh | 2011 | ******* | ↓ | very low |
| Appreciation by Volunteer Organization | − | Huynh | 2011 | ******* | ↓ | very low |
| Conflict with paid staff | + | Huynh | 2011 | ******* | ↑ | very low |
| Emotional Demands | + | Huynh | 2011 | ******* | ↑ | very low |
| Experience/time trend | + | Keith | 1997 | ** | ↑ | very low |
| Joy of volunteering | − | Huynh | 2011 | ******* | ↓ | very low |
| Motivational Screening | = | Wang | 2020 | ******* | x | very low |
| No. hours per week in hospice work | = | Keith | 1997 | ****** | x | very low |
| Organizational support | = | Huynh | 2011 | ******* | x | very low |
| Supervisor support | = | Huynh | 2011 | ******* | x | very low |
| Withdrawal | + | Huynh | 2011 | ******* | ↑ | very low |
| **Volunteer-Patient Interaction** | | | | | | |
| Volunteer-Patient Interaction | = | Huynh | 2011 | ******* | x | very low |

# Burnout

Table 4: Direction of association found in individual studies indicated as + (significant positive association), − (significant negative association), = (no significant association). Number of stars indicating quality: with *** = high quality (⩾ 70% QualSyst), **= moderate quality (50-69%). Overall association is indicated as ↑ (overall evidence for positive association), x (overall evidence for no significant association), ↓ (overall evidence for negative association).

| **Factor** | **Association** | **First author** | **Year of publication** | **Number of stars indicating quality** | **Overall Association** | **Strength of evidence** |
| --- | --- | --- | --- | --- | --- | --- |
| **Volunteer-related factors** | | | | | | |
| Age | + | Hayslip | 2021 | ******* | ↑ | low |
|  | = | Halliday | 1997 | ****** |  |  |
| Employment | + | Halliday | 1997 | ****** | ↑ | very low |
| Internal personal control | − | Hayslip | 2021 | ******* | ↓ | very low |
| Psychological health | − | Wach | 2017 | ******* | ↑ | very low |
| Self-efficacy | = | Hayslip | 2021 | ******* | x | very low |
| Sense of Locus of Control - chance | = | Hayslip | 2021 | ******* | x | very low |
| Sense of Locus of Control - internal | − | Hayslip | 2021 | ******* | ↓ | very low |
| Sense of Locus of Control - powerful others | + | Hayslip | 2021 | ******* | ↑ | very low |
| Spirituality | = | Hayslip | 2021 | ******* | x | very low |
| **Service-related** |  |  |  |  |  |  |
| Emotional demands | + | Wach | 2017 | ******* | ↑ | very low |
| Experience/time trend | − | Halliday | 1997 | ****** | ↓ | very low |
| Length of training | = | Halliday | 1997 | ****** | x | very low |
| Overall satisfaction with supervision | − | Halliday | 1997 | ****** | ↓ | very low |
| Overall satisfaction with volunteer job | − | Halliday | 1997 | ****** | ↓ | very low |
| Perceived involvement in the interdisciplinary team | − | Halliday | 1997 | ****** | ↓ | very low |
| Perceived preparation through training | − | Halliday | 1997 | ****** | ↓ | very low |
| Withdrawal | + | Hayslip | 2021 | ******* | ↑ | very low |

# Perceived stress

Table 5: Direction of association found in individual studies indicated as = (no significant association). Number of stars indicating quality: with *** = high quality (⩾ 70% QualSyst. Overall association is indicated as x (overall evidence for no significant association).

| **Factor** | **Association** | **First author** | **Year of publication** | **Number of stars indicating quality (Q)** | **Overall Association** | **Strength of evidence** |
| --- | --- | --- | --- | --- | --- | --- |
| **Service-related** | | | | | | |
| Motivational screening | = | Wang | 2020 | ******* | x | very low |

Quality assessment: Quantitative studies

|  |  | **Amenta 1984** | **Barrick 1985** | **Claxton-Oldfield 2007** | **Gillapsie 1993** | **Halliday 1997** | **Hayslip 2021** | **Huynh 2011** | **Jo 2020** | **Keith 1997** | **Lee 2019** | **Lin 2025** | **Nissim 2014** | **Paradis 1987** | **Robbins 1991** | **Robbins 1992** | **Schwerwitz 2006** | **Wach 2017** | **Wang 2020** | **Woo 2018** | **Zana 2020** |
| --- | --- | --- | --- | --- | --- | --- | --- | --- | --- | --- | --- | --- | --- | --- | --- | --- | --- | --- | --- | --- | --- |
| **1** | Question / objective sufficiently described? | Partial (1) | Yes (2) | Yes (2) | Yes (2) | Partial (1) | Yes (2) | Yes (2) | Yes (2) | Partial (1) | Yes (2) | Yes (2) | Yes (2) | Partial (1) | Yes (2) | Partial (1) | Yes (2) | Yes (2) | Partial (1) | Yes (2) | Yes (2) |
| **2** | Study design evident and appropriate? | Partial (1) | Yes (2) | Yes (2) | Partial (1) | Yes (2) | Partial (1) | Yes (2) | Yes (2) | Yes (2) | Yes (2) | Yes (2) | Yes (2) | Yes (2) | Yes (2) | Partial (1) | Partial (1) | Yes (2) | Yes (2) | Yes (2) | Yes (2) |
| **3** | Method of subject/comparison group selection or source of information/input variables described and appropriate? | Partial (1) | Partial (1) | Partial (1) | Partial (1) | Yes (2) | Yes (2) | Partial (1) | Yes (2) | Partial (1) | Yes (2) | Yes (2) | Yes (2) | Yes (2) | Partial (1) | Partial (1) | Yes (2) | Partial (1) | Yes (2) | Partial (1) | Partial (1) |
| **4** | Subject (and comparison group, if applicable) characteristics sufficiently described? | Yes (2) | Partial (1) | Yes (2) | Yes (2) | Yes (2) | Yes (2) | Yes (2) | Yes (2) | Yes (2) | Yes (2) | Yes (2) | Yes (2) | Yes (2) | Yes (2) | Partial (1) | Yes (2) | Yes (2) | Yes (2) | Yes (2) | Yes (2) |
| **5** | If interventional and random allocation was possible, was it described? | NA | NA | NA | NA | NA | NA | NA | NA | NA | NA | NA | NA | NA | NA | NA | NA | NA | NA | NA | NA |
| **6** | If interventional and blinding of investigators was possible, was it reported? | NA | NA | NA | NA | NA | NA | NA | NA | NA | NA | NA | NA | NA | NA | NA | NA | NA | NA | No (0) | NA |
| **7** | If interventional and blinding of subjects was possible, was it reported? | NA | NA | NA | NA | NA | NA | NA | NA | NA | NA | NA | NA | NA | NA | NA | NA | NA | NA | NA | NA |
| **8** | Outcome and (if applicable) exposure measure(s) well defined and robust to measurement / misclassification bias? Means of assessment reported? | Yes (2) | Yes (2) | Yes (2) | Yes (2) | Partial (1) | Yes (2) | Yes (2) | Yes (2) | Partial (1) | Yes (2) | Partial (1) | Partial (1) | Partial (1) | Partial (1) | Partial (1) | Partial (1) | Partial (1) | Partial (1) | Yes (2) | Yes (2) |
| **9** | Sample size appropriate? | Partial (1) | No (0) | No (0) | Partial (1) | Partial (1) | Partial (1) | Partial (1) | Partial (1) | Partial (1) | No (0) | Yes (2) | No (0) | Partial (1) | Partial (1) | Yes (2) | Partial (1) | Partial (1) | Partial (1) | Partial (1) | Partial (1) |
| **10** | Analytic methods described/justified and appropriate? | No (0) | Partial (1) | Partial (1) | Yes (2) | Partial (1) | Yes (2) | Yes (2) | Yes (2) | Partial (1) | Yes (2) | Yes (2) | Partial (1) | Partial (1) | Partial (1) | Yes (2) | Yes (2) | Yes (2) | Yes (2) | Yes (2) | Yes (2) |
| **11** | Some estimate of variance is reported for the main results? | No (0) | Partial (1) | Partial (1) | Yes (2) | Partial (1) | Yes (2) | Yes (2) | Yes (2) | Partial (1) | Partial (1) | Yes (2) | No (0) | Yes (2) | No (0) | Yes (2) | Yes (2) | Yes (2) | Yes (2) | Yes (2) | Yes (2) |
| **12** | Controlled for confounding? | No (0) | No (0) | No (0) | No (0) | No (0) | Partial (1) | Partial (1) | Partial (1) | No (0) | No (0) | Partial (1) | Yes (2) | No (0) | Partial (1) | Yes (2) | Partial (1) | Yes (2) | Partial (1) | Partial (1) | Partial (1) |
| **13** | Results reported in sufficient detail? | Yes (2) | Partial (1) | Yes (2) | Partial (1) | Yes (2) | Yes (2) | Yes (2) | Yes (2) | Yes (2) | Yes (2) | Yes (2) | Yes (2) | Yes (2) | Partial (1) | Yes (2) | Yes (2) | Yes (2) | Yes (2) | Yes (2) | Yes (2) |
| **14** | Conclusions supported by the results? | Yes (2) | Yes (2) | Partial (1) | Yes (2) | Yes (2) | Yes (2) | Yes (2) | Yes (2) | Yes (2) | Yes (2) | Yes (2) | Yes (2) | Partial (1) | Yes (2) | Partial (1) | Yes (2) | Yes (2) | Partial (1) | Partial (1) | Yes (2) |
|  | **Score** | 12/22 | 13/22 | 14/22 | 16/22 | 15/22 | 19/22 | 19/22 | 20/22 | 14/22 | 17/22 | 20/22 | 16/22 | 15/22 | 14/22 | 16/22 | 18/22 | 19/22 | 17/22 | 18/22 | 19/22 |
|  |  | 0.55 | 0.59 | 0.64 | 0.73 | 0.68 | 0.86 | 0.86 | 0.91 | 0.64 | 0.77 | 0.91 | 0.73 | 0.68 | 0.64 | 0.73 | 0.82 | 0.86 | 0.77 | 0.75 | 0.86 |

Quality assessment: Qualitative studies

|  |  | **Bosnjak, 2012** | **Claxton-Oldfield, 2023** | **Dein, 2005** | **Paradis, 1987** | **van den Bosch, 2023** | **Weeks, 2011** |
| --- | --- | --- | --- | --- | --- | --- | --- |
| **1** | Question / objective sufficiently described? | Yes (2) | Partial (1) | Yes (2) | Yes (2) | Yes (2) | Yes (2) |
| **2** | Study design evident and appropriate? | Partial (1) | Yes (2) | Yes (2) | Partial (1) | Yes (2) | Yes (2) |
| **3** | Context for the study clear? | Yes (2) | Yes (2) | Yes (2) | Yes (2) | Yes (2) | Yes (2) |
| **4** | Connection to a theoretical framework / wider body of knowledge? | Partial (1) | Partial (1) | Yes (2) | Yes (2) | Yes (2) | Yes (2) |
| **5** | Sampling strategy described, relevant and justified? | No (0) | Partial (1) | Partial (1) | Partial (1) | Partial (1) | Partial (1) |
| **6** | Data collection methods clearly described and systematic? | Partial (1) | Partial (1) | Yes (2) | Partial (1) | Yes (2) | Yes (2) |
| **7** | Data analysis clearly described and systematic? | Partial (1) | Yes (2) | Partial (1) | No (0) | Yes (2) | Yes (2) |
| **8** | Use of verification procedure(s) to establish credibility? | No (0) | Yes (2) | Yes (2) | No (0) | Yes (2) | No (0) |
| **9** | Conclusions supported by the results? | Yes (2) | Yes (2) | Yes (2) | Yes (2) | Yes (2) | Yes (2) |
| **10** | Reflexivity of the account? | Partial (1) | Partial (1) | Partial (1) | Partial (1) | Partial (1) | Partial (1) |
|  | **Score** | 11/20 | 15/20 | 17/20 | 12/20 | 18/20 | 16/20 |
|  |  | 0.55 | 0.75 | 0.85 | 0.6 | 0.9 | 0.8 |
